# A Pilot Study of Clinical Measures to Assess Mind-Body Intervention Effects for those with and without PTSD

**DOI:** 10.4172/2327-5162.1000116

**Published:** 2013-05-18

**Authors:** Wahbeh H, Oken BS

**Affiliations:** Department of Neurology, Oregon Health & Science University, Portland, Oregon, USA

**Keywords:** Post-traumatic stress disorder, Combat veterans, Mind-body, Heart rate variability, Cytokines

## Abstract

**Objective:**

Assess measures for future mind-body interventions in those with and without PTSD.

**Methods:**

Psychological and immune measures were assessed at baseline in three age and gender-matched groups: 1) 15 combat veterans with PTSD, 2) 15 combat veterans without PTSD, and 3) 15 non-combat veterans without PTSD. Physiological measures were assessed at baseline, during relaxation and stress conditions.

**Results:**

The PTSD group had increased PTSD and depression severity, anxiety, and mood disturbance, and decreased quality of life scores. Respiration, heart rate variability, heart rate, and blood pressure differed significantly between conditions but not between groups.

**Conclusions:**

Respiration and heart rate variability may be useful measures for future mind-body intervention trials.

## Introduction

Effective therapies are needed as increasing number of veterans return from war zones with posttraumatic stress disorder (PTSD). Up to 30.9% of male Vietnam eater veterans have had lifetime PTSD [1], and 13-17% returning Operation Enduring Freedom and Operation Iraqi Freedom soldiers screen positive for PTSD [2]. PTSD exacts high personal and societal costs. Chronic symptoms, increased psychiatric and medical co-morbidity [3] functional impairment, and mortality risk [4] drive the $45 billion estimated costs of treating PTSD and other anxiety disorders [5]. The psychological symptom complexity of PTSD is accompanied by physiological effects involving the immune, and nervous systems. PTSD treatments and outcome measures should address these multi-factorial issues. Mind-body therapies such as meditation may affect stress-related physiology [6,7]. Mind-body medicine focuses on the relationships between the brain, mind, body, and behavior, and their impact on health and disease states, and may be helpful for stress-related disorders [8]. Mind-body approaches encompass a large group of therapies including meditation, hypnosis, yoga, biofeedback, and visual imagery. Before conducting mind-body medicine randomized clinical trials, optimal measures need to be chosen.

PTSD pathophysiology alters immune and nervous system function. People with PTSD have a higher incidence of cardiovascular disease, autoimmune disease, and chronic pain, perhaps due to immune system impairment caused by chronic stress responses since some studies show increased pro-inflammatory cytokines [9-11] in individuals with PTSD. PTSD is associated with reduced heart rate variability (HRV), suggesting increased sympathetic and decreased parasympathetic tone [12]. People with PTSD are also noted to have higher resting heart rates [13] and blood pressure (BP) [14] than controls.

We anticipate successful PTSD treatment will influence multiple physiological systems. In order to generate hypotheses for future studies, we chose measures relevant to PTSD pathophysiology that are also influenced by mind-body interventions. The primary objective of this pilot study was to evaluate psychological questionnaires, HRV, heart rate, respiration, BP, cytokines, and hsCRP as potential measures for future mind-body PTSD intervention trials. Secondary aims were to assess recruitment, attendance, and adherence to collections for clinical trial planning.

## Methods and Materials

### Participants

Potential participants were recruited through flyers at the Portland Veterans Administration Medical Center, Portland Veterans Center, and other veterans groups throughout the Portland Metropolitan area. The three age and gender-matched participant groups were 15 combat veterans with PTSD, 15 combat veterans without PTSD, and 15 non-combat veterans without PTSD. Veterans were excluded if they were over the age of 70, had a current significant chronic medical illness, bipolar, schizoaffective, or psychotic disorders; any DSM-IV cognitive disorder; substance dependence disorder within 3 months of the study or current substance use other than alcohol (≤ 2 drinks/day); or sexual assault as primary PTSD event/s. The participants needed to be on stable doses of medications for at least 4 weeks prior to study onset. The study was approved by the institutional review boards of Portland Veterans Administration Medical Center and Oregon Health & Science University. Written informed consent was obtained from all subjects.

### Clinical assessments

Potential subjects were interviewed by trained clinicians using the Clinician-Administered PTSD Scale for DSM-IV (CAPS) [15] to validate the presence or absence of PTSD. Participants in the PTSD group met DSM-IV-TR criteria A-F; criteria B, C, and D were met when the frequency plus intensity score were ≥ 4. Participants in the non-PTSD groups did not meet full syndrome criteria. The Structured Clinical Interview for DSM-IV-Patient Edition (SCID-IV) was performed by the same clinicians to screen for excluded DSM-IV disorders [16]. Combat exposure was determined with the Combat Exposure Scale (CES) [17].

### Psychological assessments

All questionnaires used were validated instruments previously implemented in clinical trials: *Posttraumatic Stress Disorder Checklist Scale* (*PCL*) [18], *Beck Depression Inventory* (*BDI*) [19], *Perceived Stress Scale* (*PSS*) [20], *State Trait Anxiety Inventory* (*STAI*) [21], *Profile of Mood States* (*POMS*) [22], and *SF-36^®^ Health Survey* (*SF-36*) [23].

### Study procedures

**Figure 1:**
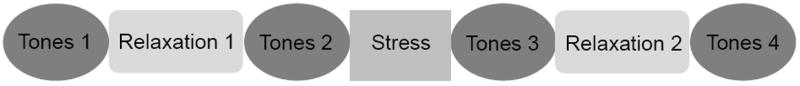
Laboratory Conditions: At the laboratory visit, participants experienced three conditions: Tones, Relaxation and Stress in the order represented above. Participants were sitting for approximately 30 minutes while physiological monitoring devices were connected.

Study procedures, inclusion/exclusion criteria, and the risks and benefits of participating were explained during telephone screening. The screening visit included informed consent procedures, CAPS and SCID administration, and CES and PCL completion. Eligible participants were given questionnaires to complete at home and a home saliva collection kit. Electrocardiogram (ECG), BP, and respiration were recorded during several conditions at the laboratory visit (Figure 1). Task stimuli for all conditions were created and presented in EPrime 2.0 (Psychology Software Tools, Inc., Pittsburgh, PA).

### Tones

The four minute Tones task provided an eyes closed baseline where the participant remained alert and awake. The participant sat with eyes closed with a trigger button in each hand and would click the right button after hearing a high tone (2000 Hz) and the left button after hearing the low tone (1000 Hz). The tones were presented prior to initiating the task to familiarize participants with and check their ability to distinguish between them. The tones were randomly presented for one second with a random and variable interval between the tones (4-14 sec).

### Relaxation

The 13 minute relaxation task consisted of paced breathing with the Food and Drug Administration approved RESPeRATE device [24] (Intercure, Inc., New York, NY), which consists of a microprocessor, respiration sensor, and headphones that provide feedback to the participant. The system registers the participant’s breathing rate and personalizes a melody with two tones that corresponds to inspiration and expiration. The participants were seated with the headphones on, and the respiration sensor elastic around their chest. They inhaled during one tone and exhaled during the other tone. The tones began at the participant’s current rate then gradually slowed to achieve ten breaths per minute (bpm) or less. During this task, participants watched 162 neutral nature scene pictures (without people) that were randomly displayed for four seconds with a one second inter-stimulus interval. These pictures helped participants keep their eyes open and matched the stressor’s presentation format. After Relaxation 1 and Relaxation 2, the participant was asked to rate subjective relaxation on a scale of 1-10, with 10 being the most relaxed.

### Stressor

The 13 minute stressor condition consisted of viewing 162 aversive pictures from the International Affective Picture System (IAPS) (low pleasure (valence mean=2.1), high arousal (arousal mean=5.8), high dominance (dominance mean=3.8) [25]. The IAPS includes emotionally-evocative color photographs known to affect heart rate, skin conductance, startle reflex, and brain activation in emotional processing areas. Pictures were shown for four seconds with a one second inter-stimulus interval. In order to ensure participant attention, they were asked to click the right hand button every time they saw a snake. After the stressor condition, the participant was asked to rate subjective stress on a scale of 1-10, with 10 being the most stressed.

## Physiological Data

ECG data was collected with electrodes placed bilaterally just inferior to the clavicles and was digitized at 1024 samples per second using BioSemi amplifiers (Active 2, Biosemi, Amsterdam, Netherlands). Waves were detected automatically and beat-to-beat intervals extracted using Brain Vision Analyzer 2.0 (Brain Products GmbH, Inc., Gilching, Germany). R intervals with erroneous detections or aberrant beats were eliminated by visual inspection off-line. Mean heart rate was calculated by taking the average of the normal inter-beat intervals. RR was calculated as the variance of the normal-normal beat (NN) interval for each condition. Respiration rate was measured with a light elastic piezoelectric belt (Ambu-Sleepmate, Maryland) around the participant’s chest near the diaphragm, recorded using BioSemi, and counted for each condition. BP was recorded with an inflatable finger cuff monitor (Finapres, Ohmeda, Madison, WI), placed on the middle finger of the left hand and inflated every 15 seconds. Peak and trough amplitudes were averaged for each condition and converted to mmHg values using a Finapres conversion formula. Because the Relaxation and Stress conditions were longer than the Tones, data segments to match Tones were extracted from the middle of the longer conditions for BP, heart rate, and HRV.

## Biological Samples

A blood draw was performed after the laboratory visit, between 11:30 am and 2:30 pm. Twenty milliliters of blood were collected from each subject for IL-6, TNF-α, and hsCRP. Plasma (~10 mls) was separated, divided into 1 ml aliquots, and stored at -80°C. All samples were quantified by the Oregon Clinical and Translational Research Institute lab including cytokines in duplicate using high sensitivity ELISA (R&D Systems Laboratory, Minneapolis, MN) and hsCRP by an immulite platform chemiluminescent assay system (Siemens Medical Solutions Diagnostics, Los Angeles, CA).

## Statistical Analyses

All statistical analyses were performed with SPSS 17.0 (SPSS, Chicago, IL, USA). Power analysis for a test of the null hypothesis was conducted with Sample Power 1.2 (SPSS, Chicago, IL, USA) *a priori* using a two-tailed fixed effects analysis of covariance (ANCOVA) model which determined sample size. Data were examined for outliers and normality of distribution. Natural log and square root transformations were performed as needed. Group differences for questionnaire and demographic data were assessed with one-way analysis of variance and the chi-square test. Baseline group differences (Tones 1) for physiologic measures were assessed with ANCOVA including the covariates age (continuous), bpm (continuous), and anti-hypertensive medications (categorical) in the model where appropriate. Group differences were assessed using depression, sleep, and smoking, BMI, and clock time as covariates. If a covariate was not significant, it was removed from the model and the ANCOVA was repeated with the simplified model. For repeated physiological measures, differences were assessed using a mixed model linear approach with group and condition as fixed factors and covariates included as described above. In this exploratory analysis, an alpha of 0.05 was used as statistically significant without correction for multiple comparisons. Using a Holm-Bonferroni correction yielded an alpha of 0.002 for significance [27]. Pearson *r* was calculated to assess correlations between measures.

## Results

### Feasibility

Seventy-seven people were telephone screened. Fifty (65%) were eligible and enrolled. Three subjects screened out at the first visit. Two control subjects could not be matched with the PTSD group and were dropped from the analysis. There was 100% participation in all study activities of the remaining 45 male veterans. A few biological samples were missing due to blood draw failure (3). No adverse events were reported.

### Demographics

ere were no group differences on age, race, education, marital status, exercise, sleep, handedness, past psychiatric history, past major depressive disorder, antidepressant use, past or current drug, alcohol, or cigarette use, and medications affecting ECG or BP (*ps*>0.05) (Table 1). Era differed between groups as expected, with mostly Vietnam veterans in the PTSD group and “Other” in the non-combat, non-PTSD group (*X*^2^=10.4, *p*=0.03), as did combat exposure (*F*(2,44)=54.0; *p*=<.0005), and years in military service (*F*(2,44)=1.24, *p*=.03).

### Psychological data

As expected, CAPS and PCL scores reflected greater PTSD severity in the PTSD group (CAPS: *F*(2,44)=120.03, *p*<0.0005; PCL: *F*(2,44)=30.42, *p*<0.0005) (Table 2). The PTSD group was more likely in a current major depressive episode (*X^2^*=6.42, *p*=0.04), and had more severe depression symptoms (BDI: *F*(2,44)=3.20, *p*=0.05; POMS-Depression: *F*(2,44)=4.03, *p*=0.03). Trait anxiety (STAI-T: *F*(2,44)=4.45, *p*=0.02), perceived stress (PSS: *F*(2,44)=3.47, *p*=0.04), and tension (POMS-Tension: *F*(2,44)=6.62, *p*=0.003) were higher, and SF36 emotional role (*F*(2,44)=3.6, *p*=0.04) and mental health (*F*(2,44)=6.28, *p*=0.004) subscale scores were lower in the PTSD group. During the stress condition, the PTSD group reported the most subjective stress (5.3 ± 2.3), followed by the non-combat non-PTSD group (4.4 ± 2.0), with the combat non-PTSD group (3.1 ± 2.0) reporting the least subjective stress (*F*(2,44)=4.30, *p*=0.02). No other self-report questionnaires showed significant between-group differences (*ps*>0.05).

### Physiological data

**Figure 2:**
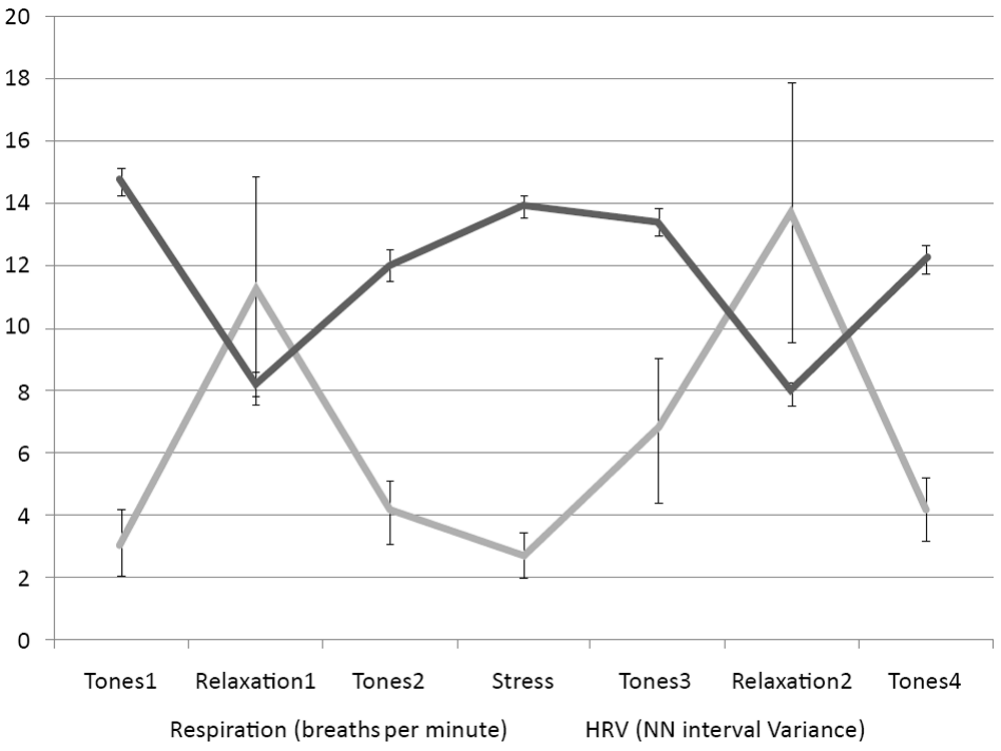
Respiration and HRV across all conditions: Respiration (bpm) and HRV (normal-normal inter-beat variance for each condition) are shown with standard errors of the mean at each point. Note the expected increased variability in HRV with slow deep breathing during Relaxation1 and Relaxation2.

At baseline and within conditions, there were no group differences in respiration, BP, HRV, or heart rate (*ps*>0.05) (Table 3). The main effect of condition was significant for respiration (*F*(6,41)=39.4, *p*<0.0005), HRV, heart rate, and systolic and diastolic BP. Extra period before (Figure 2). Also, the effect of Condition was different for HRV in models including all conditions (*F*(6,43)=12.8, *p*<0.0005). Further analyses co-varying for respiration rate, age, and medications remained significant. Heart rate increased from baseline during the relaxation conditions and remaining unchanged during the stress condition (*F*(6,43)=14.8, *p*<0.0005). Systolic BP decreased during the relaxation conditions and remained the same for the stress condition (relative to the previous baseline) (*F*(6,41)=6.6, *p*<0.0005) (Table 4). Diastolic BP increased during Relaxation and decreased during Stress (relative to the previous baseline) (*F*(6,43)=3.8, *p*=0.004).

### IL-6, TNF-α, hsCRP

There were no group differences for IL-6, TNF-α, or hsCRP (*ps*>.05) (Table 5).

## Discussion

### Study groups

The three study groups were matched on age, gender, and other demographic measures. Women were recruited for this study, but none were eligible due to reporting military sexual rather than combat trauma as the primary trauma. The decision to reduce heterogeneity in our participant pool by only including combat trauma as the primary PTSD event was necessary with our small sample size. Military sexual trauma is clearly an important clinical and research area as more women serve in the military and are reported to experience more military sexual trauma than men [28,29].

Additionally, most of the participants were Vietnam veterans. Almost 30 years after Vietnam, 10% of veterans continue to experience severe PTSD [30]. PTSD was not a diagnosis when Vietnam veterans returned. Many older veterans now understand that they have or have had PTSD due to increased popular awareness of PTSD. Vivid images of Iraq and Afghanistan often trigger their PTSD symptoms motivating them to seek treatment. Reaching younger veterans from the Iraq and Afghanistan conflicts is often challenging due to the stigma of being labeled with a mental illness, not wanting to get help through the Veterans Administration or otherwise, and not being connected through veteran service organizations such as Veterans of Foreign Wars and the American Legion [31].

### Psychological data

As expected, questionnaire data validated group assignment with the PTSD group reporting increased PTSD symptoms, depression, anxiety, mood disturbance, and reduced mental health and emotional role quality of life measures. The PTSD group also reported greater subjective stress during the stressor compared to both the combat and non-combat control groups.

### Heart Rate, HRV, respiration, BP

The lack of findings between groups at baseline and across conditions was unexpected, as previous studies reported differences in heart rate [32,33] HRV [34], and BP [32] that correlated with PTSD status. However, some studies have also reported negative findings [35]. One meta-analysis found significant effect sizes for changes in heart rate, systolic and diastolic BP with trauma-related stimuli under laboratory conditions, but no differences in resting measures. Also, during stressful conditions, not all studies detected differences between PTSD subjects and controls [32]. The negative findings in our study may be due to the small sample size, or other predictors that were not assessed.

The difference of respiration and HRV across conditions was encouraging for their use in observing changes over time in participants from mind-body interventions. NN interval HRV method was appropriately responsive to changes in condition even when co-varying for respiration rate. NN interval HRV does not rely on Fast Fourier Transform which may lead to misleading results in the low frequency HRV measure if a participant is breathing slowly. At respiration rates of nine or lower, the normal sinus arrhythmia generates heart rate frequencies below the commonly used cut point of low frequency HRV, 0.15 Hz, increasing the value. There is currently no ideal way to deal with this phenomenon and yet, it is important for researchers to at least record and report breathing rate as a potential confounder in studies with HRV.

Heart rate and BP were responsive to condition, but the direction of change was contrary to the expected direction of heart rate decreasing with relaxation and increasing with stress. Heart rate actually increased with both relaxation conditions. In post-hoc analyses, we found no significant correlations between self-reported relaxation and stress scores and heart rate for each condition. There have been reports of lack of physiological response of PTSD patients in laboratory stressor settings [36,37]; however, our study included both PTSD and non-PTSD participants.

Many factors such as perception and mood may have affected our results. The relaxation task may have been “stressful” in that it was novel and required some adaptation of breathing. The stressor may have been strongly anticipated as the participants were primed through the telephone screening, consent procedures, and before the laboratory visit about the stressor. Co-morbid depression was not an exclusion because of its high prevalence in this population and may have influenced results. However, adjusting for depression scores did not change the results. There may also be a common physiological conditioning in veterans based on military training that diminishes detectable changes during stressful laboratory conditions. Another potential issue was carryover effect between conditions. We attempted to minimize this effect by including the Tones task between each condition. Despite the lack of group difference on physiological measures, the PTSD group subjectively rated the stressor as more stressful than the other groups. Either there was dissociation between physiological and subjective stress, or the study was not adequately powered to show a small difference between groups.

### IL-6, TNF-α, hsCRP

Some studies have found higher levels in IL-6 [9,38,39], TNF-α [11,40], and hsCRP [41] in PTSD, while others did not [10,39]. However, only one of these studies assessed veterans using dexamethasone administration and LPS-stimulated whole-blood culture [40].

In summary, there were no issues with recruitment, retention and compliance encouraging future clinical trials in this population. HRV and respiration showed changes in all groups in response to relaxation and stress and could be useful measures of compliance or response in future studies. Heart rate and BP also changed with condition and may be used with caution to track within-subject changes in an intervention study. The cytokines and hsCRP need further research before they can be reliably used as a measure in PTSD intervention studies.

## Figures and Tables

**Table 1:** Demographic data.

Characteristic	Combat, PTSD (n=15)	Combat, Non-PTSD (n=15)	Non-Combat, Non-PTSD (n=15)	Statistics

Exercise% None	2 (13)	2 (13)	1 (7)	*X^2^*=3.25, *p*=0.80
1-2x/week	2 (13)	2 (13)	2 (13)	
3-4x/week	3 (20)	4 (27)	1 (7)	
4-5x/week	8 (53)	9 (60)	11 (73)	

Sleep<4	1 (7)	1 (7)	1 (7)	*X^2^*=7.8, *p*=0.10
5-7	11 (73)	11 (73)	7 (47)	
8-10	3 (20)	3 (20)	7 (47)	

Past Drug Use True	8 (53)	6 (40)	6 (40)	*X^2^*=0.72, *p*=0.70

Past Alcohol use True	12 (80)	11 (73)	11 (73)	*X^2^*=0.24, *p*=0.89

Current Alcohol use True (<2dr/day)	4 (27)	4 (27)	5 (33)	*X^2^*=0.22, *p*=0.90

Past smoking True	12 (80)	11 (73)	6 (40)	*X^2^*=1.51, *p*=0.47

Current Smoking True	3 (20)	6 (40)	6 (40)	*X^2^*=1.80, *p*=0.41

Antidepressant use	6 (40)	2 (13)	5 (33)	*X^2^*=2.81, *p*=0.25

Past major depressive disorder	7 (47)	5 (33)	7 (47)	*X^2^*=0.73 *p*=0.70

Current major depressive disorder	3 (20)	0 (0)	0 (0)	*X^2^*=6.43, *p*=0.04*

Medications that affect ECG true statistics column	3 (20)	2 (14)	4 (27)	*X^2^*=0.83, *p*=.66

Medications that affect BP True	3 (20)	1 (7)	3 (20)	*X^2^*=1.35, *p*=0.51

Demographic data is presented for exercise, sleep, past drug use true, past alcohol use true, current alcohol use true (less than two drinks per day), past smoking true, current smoking true, antidepressant use, and past and current major depressive disorder in number per group and percent n (%).

PTSD=Post-Traumatic Stress Disorder. ECG=Electrocardiography. BP=Blood Pressure.

Statistics are for Chisquare test by group. There were no significant differences between groups on any of these measures except for current major depressive disorder with the PTSD group being more likely to have the disorder.

* =statistically significant without multiple comparisons correction

**Table 2:** Psychological data.

Characteristic	Combat, PTSD (n=15)	Combat, Non-PTSD (n=15)	Non-Combat, Non-PTSD (n=15)	Statistic
CAPS	75.3 ± 13.2	11.6 ± 11.8	10.9 ± 4.1	*F*(2,44)=120.03, *p*<0.0005**
PCL	58.7 ± 11.3	28.5 ± 8.5	32.0 ± 14.3	*F*(2,44)=30.42, *p*<0.0005**
BDI	19.8 ±13.7	9.8 ± 8.0	13.0 ± 9.4	*F*(2,44)=3.2, *p*=0.05*
State Anxiety	44.7 ± 11.1	36.0 ± 13.9	40.9 ± 14.4	*F*(2,44)=1.63, *p*=0.21
Trait Anxiety	50.8 ± 13.0	38.2 ± 10.1	43.7 ± 11.5	*F*(2,44)=4.45, *p*=0.02*
Perceived Stress Scale	22.3 ± 6.3	16.7 ± 7.1	16.3 ± 7.3	*F*(2,44)=3.47, *p*=0.04*
Combat Exposure Scale	29.6 ± 10.1	18.9 ± 8.8	0.5 ± 1.1	*F*(2,44)=53.97, *p*<0.0001**
POMS Tension	24.1 ± 7.6	16.4 ± 4.6	19.7 ± 5.2	*F*(2,44)=6.62, *p*=0.003*
POMS Depression	34.5 ± 14.2	23.4 ± 8.4	28.5 ± 11.0	*F*(2,44)=4.03, *p*=0.03*
POMS Anger	26.4 ± 10.1	20.7 ± 10.0	22.1 ± 8.2	*F*(2,44)=1.46, *p*=0.24
POMS Vigor	20.7 ± 7.6	23.6 ± 6.4	25.0 ± 8.9	*F*(2,44)=1.34, *p*=0.27
POMS Fatigue	16.5 ± 7.4	15.5 ± 7.0	14.1 ± 5.7	*F*(2,44)=0.49, *p*=0.62
POMS Concentration	18.3 ± 5.3	14.7 ± 3.2	15.1 ± 4.1	*F*(2,44)=2.9, *p*=0.07
POMS Total	99.3 ± 44.0	67.1 ± 32.0	74.6 ± 36.6	*F*(2,44)=2.94, *p*=0.06
Relaxation 1	7.3 ± 1.4	7.6 ± 1.4	7.9 ± 1.3	*F*(2,44)=0.91, *p*=0.41
Stress	5.3 ± 2.3	3.1 ± 2.0	4.4 ± 2.0	*F*(2,44)=4.30, *p*=0.02*
Relaxation 2	7.3 ± 1.7	7.7 ± 1.5	7.9 ± 1.4	*F*(2,44)=0.47, *p*=0.63
Mindful Awareness	41.7 ± 12.3	47.1 ± 10.4	40.5 ± 11.1	*F*(2,44)=1.44, *p*=0.25
Mindful Non-Judging	22.5 ± 8.2	33.5 ± 7.1	30.7 ± 9.4	*F*(2,44)=7.22, *p*=0.002**
SF36 Physical Function	51.1 ± 15.8	55.6 ± 15.3	53.6 ± 14.8	*F*(2,44)=0.32, *p*=0.73
SF36 Physical Role	49.0 ± 22.3	61.0 ± 21.7	56.3 ± 24.0	*F*(2,44)=1.06, *p=*0.35
SF36 Emotional Role	43.1 ± 22.9	61.3 ± 16.8	60.0 ± 22.0	*F*(2,44)=3.6, *p*=0.04*
SF36 Vitality	37.7 ± 19.1	44.3 ± 18.7	46.7 ± 16.0	*F*(2,44)=1.01, p=0.37
SF36 Mental Health	37.9 ± 16.3	55.2 ± 13.8	50.9 ± 11.4	*F*(2,44)=6.28, *p*=0.004*
SF36 Social Function	42.0 ± 22.1	60.7 ± 25.5	54.7 ± 18.1	*F*(2,44)=2.79, *p*=0.07
SF36 Pain	46.3 ± 21.1	59.7 ± 18.9	52.2 ± 24.9	*F*(2,44)=1.42, *p*=0.25
SF36 General Health	44.8 ± 11.9	51.5 ± 14.0	50.7 ± 10.0	*F*(2,44)=1.36, *p*=0.27

The mean and standard deviation for each psychological measure are listed by group with statistics for analysis of covariance.

PTSD-post-traumatic stress disorder; CAPS-Clinician Administered PTSD Scale; PCL-PTSD checklist; BDI-Beck Depression Inventory; POMS-Profile of Mood States; SF36-SF36 Health Survey.

* =statistically significant without multiple comparisons correction,

** =statistically significant with multiple comparison correction

**Table 3:** Physiological data.

Measure	Respiration	HRV	Heart Rate	Systolic BP	Diastolic BP

Tones1	14.7 ± 3.3	3.1 ± 8.3	70.5 ± 10.4	117.3 ± 6.1	65.8 ± 12.3

Relaxation 1	8.2 ± 3.0	11.2 ± 28.2	71.2 ± 9.7	115.7 ± 4.5	67.6 ± 11.5

Tones2	12.0 ± 4.0	4.1 ± 7.7	69.4 ± 9.4	117.2 ± 5.9	66.1 ± 11.6

Stress	13.9 ± 2.8	2.7 ± 5.7	69.5 ± 10.0	116.9 ± 7.3	60.8 ± 14.2

Tones3	13.4 ± 3.4	6.7 ± 17.9	69.3 ± 9.4	118.6 ± 6.7	62.5 ± 11.7

Relaxation 2	7.9 ± 2.9	13.7 ± 32.2	71.0 ± 9.6	117.4 ± 5.0	63.8 ± 11.0

Tones4	12.2 ± 3.5	4.2 ± 7.7	68.7 ± 9.4	118.5 ± 5.8	63.5 ± 11.7

All Conditions	*F*(6,41)=39.4	*F*(6,43)=12.8	*F*(6,43)=14.8	*F*(6,41)=6.6	*F*(6,43)=3.8
	*p*<0.0005**	*p*<0.0005**	*p*<0.0005**	*p*<0.0005**	*p*=0.004*

The mean and standard deviation for respiration, HRV (Heart Rate Variability), heart rate, systolic and diastolic BP (Blood Pressure) are listed by condition with statistics.

* =statistically significant without multiple comparisons correction,

** =statistically significant with multiple comparison correction

**Table 4:** Physiological Data by Group.

Measure	Respiration			HRV			Heart Rate			Systolic BP			Diastolic BP		
Group	C, +	C, -	NC, -	C, +	C, -	NC, -	C, +	C, -	NC, -	C, +	C, -	NC, -	C, +	C, -	NC, -
Tones1	14.77 (2.93)	14.68 (3.64)	15.55 (3.34)	3.14 (8.42)	3.9 (10.55)	2.05 (3.73)	69.74 (7.01)	71.24 (11.78)	70.52 (10.58)	118.39 (5.21)	116.19 (5.79)	117.39 (7.45)	66.02 (13.43)	63.48 (11.01)	66.39 (14.91)
Relaxation 1	7.15 (1.44)	8.31 (3.55)	9.47 (3.74)	8.61 (11.22)	17.32 (45.74)	5.41 (6.47)	72.15 (7.52)	73.17 (10.97)	71.1 (9.93)	116.32 (4.49)	115.1 (5.34)	116.3 (5)	65.48 (16.04)	68.27 (10.22)	65.95 (13.07)
Tones2	10.78 (2.87)	12.23 (5.06)	12.99 (3.43)	3.3 (5.52)	5.93 (10.83)	2.42 (4.8)	70.19 (6.51)	69.5 (10.57)	68.77 (10.26)	118.12 (5.54)	115.99 (6.21)	117.38 (6.87)	66.43 (14.65)	65.38 (9.87)	64.97 (12.21)
Stress	13.65 (2.71)	13.83 (3.44)	14.71 (2.07)	1.89 (2.67)	4.57 (8.53)	1.27 (3.08)	70 (8.42)	69.11 (10.4)	69.06 (10.38)	119.15 (7.56)	115.51 (6.35)	116.65 (7.77)	56.19 (18.38)	62.82 (11.98)	61.18 (13.02)
Tones3	13.03 (2.86)	13.75 (4.24)	13.9 (3.05)	3.63 (4.82)	10.64 (27.12)	4.74 (11.99)	69.95 (7.68)	69.39 (10.75)	69.39 (9.62)	119.64 (5.77)	115.94 (6.06)	119.08 (8.99)	59.44 (14.84)	64.77 (12.31)	63.78 (10.94)
Relaxation 2	6.88 (1.18)	8.86 (4.43)	8.34 (2.76)	11.56 (19.11)	18.96 (48.06)	9.09 (19.39)	70.68 (7.03)	71.81 (11.66)	69.85 (9.02)	119.07 (5.55)	116.08 (5.01)	117.28 (4.51)	61.09 (15.98)	66.26 (9.6)	62.57 (10.97)
Tones4	11.06 (2.79)	12.88 (3.88)	12.75 (3.52)	3.37 (3.9)	4.95 (9.43)	5.78 (11.15)	68.83 (6.92)	68.78 (10.62)	68.08 (9.63)	119.09 (6.07)	116.06 (5.56)	119.74 (6.33)	61.92 (15.43)	64.63 (9.13)	62.86 (12.29)

The mean and standard deviation for respiration, HRV (Heart Rate Variability), heart rate, systolic and diastolic BP (blood pressure) are listed by condition and by group. Mean (standard deviation).

C=Combat, NC=No-combat, +=PTSD, -=No PTSD

**Table 5:** IL-6, TNF-α, hsCRP Data.

Measure	Combat, PTSD (n=15)	Combat, Non-PTSD (n=15)	Non-Combat, Non-PTSD (n=15)	Statistic
IL-6 (pg/mL)	1.62 ± .69	1.71 ± .79	2.30 ± 2.48	*F*(2,41)=0.35, *p*=0.89
TNF-α (pg/mL)	1.76 ± .35	1.92 ± .31	2.71 ± .29	*F*(2,41)=1.68, *p*=0.20
hsCRP (mg/L)	4.12 ± 6.72	2.72 ± 3.71	3.25 ± 5.53	*F*(2,41)=0.87, *p*=0.43

The mean and standard deviation for IL-6, TNF-α, and hsCRP are for each group.

PTSD-Post-Traumatic Stress Disorder; IL-6-interleukin 6; TNF-α-Tumor Necrosis Factor Alpha; hsCRP-highly sensitive C-Reactive Protein
